# Characterization of a New Dry Drill-Milling Process of Carbon Fibre Reinforced Polymer Laminates

**DOI:** 10.3390/ma11081470

**Published:** 2018-08-18

**Authors:** Alessandra Caggiano, Ilaria Improta, Luigi Nele

**Affiliations:** 1 Fraunhofer Joint Laboratory of Excellence on Advanced Production Technology (Fh-J_LEAPT UniNaples), 80125 Naples, Italy; alessandra.caggiano@unina.it; 2 Department of Industrial Engineering, University of Naples Federico II, 80125 Naples, Italy; 3 Department of Chemical, Materials and Industrial Production Engineering, University of Naples Federico II, 80125 Naples, Italy; ilaria.improta@unina.it

**Keywords:** CFRP, hole making, dry machining, roughness

## Abstract

Carbon Fibre Reinforced Polymer (CFRP) composites are widely used in aerospace applications that require severe quality parameters. To simplify the assembly operations and reduce the associated costs, the current trend in industry is to optimize the drilling processes. However, the machining of CFRP composites is very challenging compared with metals, and several defect types can be generated by drilling. The emerging process of orbital drilling can greatly reduce the defects associated with the traditional drilling of CFRP, but it is a more complex process requiring careful process parameters selection and it does not allow for the complete elimination of the thrust force responsible for delamination damage. As an alternative to traditional and orbital drilling, this work presents a new hole making process, where the hole is realized by a combination of drilling and peripheral milling performed using the same cutting tool following a novel tool path strategy. An original tool design principle is proposed to realize a new drill-milling tool, made of a first drilling and a subsequent milling portion. Two different tool configurations are experimentally tested to evaluate the performance of the newly-conceived combined drill-milling process. This process is quick and easy, and the experimental results show an improvement in the drilled hole quality.

## 1. Introduction

Carbon fibre reinforced polymer (CFRP) composites are widely used in the aerospace industry. In this sector, the prevalent machining process performed on CFRP components is drilling, as mechanical joining by means of rivets or bolts represents the most common joining technique for aircraft components. However, the drilling of CFRP is very challenging in comparison with the drilling of metals, as the phenomena underlying the material removal for composite materials are substantially different from those characteristic of metal machining [1]. The different properties of the reinforcement and matrix phases make the material removal mechanism highly complex, because of the heterogeneity and anisotropic behaviour of composites [1,2,3]. König [4] highlighted how the machining of composite materials depends on the specific properties and relative content of the reinforcement and matrix. Moreover, when machining fibre reinforced composite materials, fibre orientation plays a fundamental role, affecting the mechanism of chip formation and the cut surface quality. During the drilling process, because of the heterogeneous and anisotropic behaviour of the fibre reinforced composites, different kinds of damage can be generated in the workpiece, such as delamination, fibre pull-out, fibre breakage, matrix cracking, and thermal damage [5,6], which can affect the drilled component service life. The most relevant damage induced during CFRP drilling is delamination [7,8], which has been considered as the principal cause of notable reduction in the fatigue strength of composite components, cutting down the long-term performance of the CFRP parts. In particular, delamination onset at the hole exit, also known as push-out delamination, is generated if the axial load exerted on the workpiece during drilling exceeds a threshold value [9]. As demonstrated by Davim and Reis [10], the delamination damage grows with increasing cutting speed and feed values. As a matter of fact, a feed rate increase raises the thrust force, with consequent delamination damage enlargement at the hole exit [11].

Moreover, tool geometry significantly affects the drilling induced damage [10,12]. Designing new tools with a better performance in terms of cost, damage reduction, and hole quality is a key factor for drilling process optimisation [12]. Piquet et al. [13] analysed the effects of drilling tool geometry, comparing the results obtained with a traditional tool and a specially designed cutting tool. Feito et al. [14] and Gaitonde et al. [15] demonstrated how the delamination increases with increasing the point angle of the twist drill bit for high speed drilling of CFRP laminates. Saoudi et al. [16] developed a model to predict the critical thrust force responsible for exit-ply delamination during the drilling of multi-directional carbon fibre-reinforced plastic laminates with core drills made of diamond grits. Cadorin et al. [17] studied the mechanisms of damage and tool wear in the drilling of three-dimensional (3D) woven composite materials using diamond-coated carbide three lips twist drills. Zitoune et al. [18] investigated the influence of the tool coating on temperature and tool wear when drilling 3D woven composite materials using three types of cutting tools, one coated with a diamond layer and the other two coated with different nano-composite multi-layers, showing that the nature of the coating is a key factor affecting the temperature of machining.

To reduce the delamination and thermal damage defects generated when drilling composites, a new process called orbital drilling (OD) has been proposed in the literature. This process is more versatile than traditional drilling, as it allows for obtaining holes with different diameters without replacing the tool. OD is a circular ramp machining process, consisting of milling with a discontinuous peripheral cut and drilling with a continuous cut along the cutting-edge axis at the same time. Hence, this technology involves the simultaneous movement along a circular path (*X*, *Y*) and an axial advancement (*Z*) with a pre-determined step [19].

In OD, the orthogonal thrust exerted on the surface is very low, resulting in a superior hole quality, especially in the case of the composite materials subject to delamination damage. This type of machining process is particularly suitable for applications in the aeronautical field, as it allows for a decrease of cutting forces and temperatures, which results in a reduction of polymer matrix damage [20]. On the other hand, the main drawbacks related to OD concern the difficulties in selecting the proper process parameters and the need to employ a three-axis machining centre to avoid vibration problems. OD operations are carried out by portable and highly flexible machines, which tend to exhibit severe chatter and forced vibrations that lead to a poor hole quality. Moreover, the thrust force responsible for delamination is reduced but still not eliminated with OD [21]. As a matter of fact, while in traditional drilling, the axial loading consists of a concentrated load at the centre of the last ply of the laminate, in orbital drilling, the axial loading is determined by an eccentric distributed load, which acts along the cutting-edge of the mill [20].

As an alternative to both traditional drilling and orbital drilling processes, this research work proposes an innovative hole making process for CFRP components, where the hole is realised by a combination of drilling and peripheral milling, carried out using the same cutting tool. The objective is to further reduce the process-induced delamination damage in comparison with traditional and orbital drilling. To perform the new combined drill-milling process, an innovative drill-milling tool was developed. The main results of the experimental drill-milling tests on the CFRP composite laminates are reported in this paper, showing encouraging results in terms of the hole surface finish. The new drill-milling process is performed under dry conditions in a green technology perspective, offering advantages because of the absence of cutting fluids and a lower environmental impact.

## 2. Proposed Drill-Milling Process

In conventional drilling of fibre reinforced composite materials, in accordance with the model of linear elastic fracture mechanics, the delamination onset at the hole exit, also known as push-out, occurs if the axial load acting on the workpiece exceeds a threshold value [9,22]. The axial load performs a work equal to the sum of the energy necessary to deform the last ply of the laminate and the energy required to generate a new fracture surface [23].

**Figure 1:**
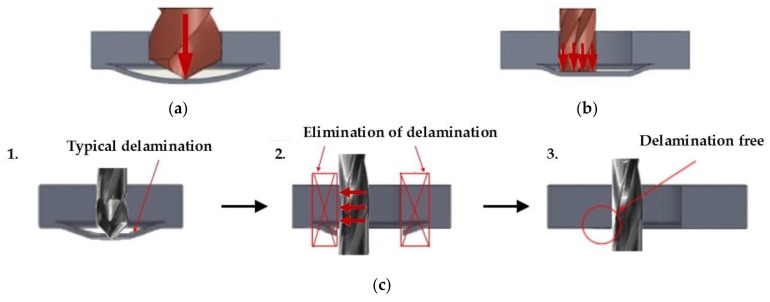
Representation of (**a**) traditional drilling; (**b**) orbital drilling [20] and (**c**) drill-milling processes. The red arrows indicate the load.

For traditional drilling, the axial load evaluation generally refers to a concentrated load at the centre of the last ply of the laminate, as studied in the delamination analysis by Hocheng et al. [9,24]. Instead, for orbital drilling, an energy criterion characterized by the application of an eccentric distributed load, which acts along the cutting-edge of the mill, is adopted. This means that the thrust force causing delamination is reduced but not eliminated, as reported in Figure 1a,b [20].

To fully eliminate the drilling-induced delamination, a different original technique for CFRP hole making is proposed in this work, where the hole is realised by means of a combination of drilling and peripheral milling processes. In Figure 1c, the two phases of the process are illustrated, namely: in the first phase traditional drilling is employed to produce a hole with a smaller diameter than the final hole, and then, in the second phase, the material around the first hole is removed by peripheral milling until the final diameter is achieved. These two phases are sequentially combined in a single process carried out using the same cutting tool.

To develop this technique, an innovative drill-milling tool was designed and realized, and experimental cutting tests on CFRP laminates were performed to characterize the process. During the machining tests, the thrust force and radial force signals were detected through a sensor system to allow for the analysis of the cutting forces occurring during the process. Moreover, the produced holes were characterised via metrological and roughness measurement procedures to evaluate the hole quality in terms of size, roundness, and internal surface finish.

## 3. Materials and Methods

### 3.1. Preparation of the Specimen

The work-material employed for the drill-milling tests consisted of CFRP laminates composed of 26 prepreg plies, made of CYCOM 977-2, Cytec Industries Inc., Woodland Park, NJ, USA epoxy matrix, and Toray T300 unidirectional carbon fibres (Toray Industries, Inc., Tokyo, Japan), with stacking sequence [±45/0/±45/90/±45/0/−45/90/45/90]_s_. The main mechanical properties of the single unidirectional plies are reported in Table 1.

The nominal thickness of each CFRP laminate was 5 mm, and a thin fiberglass/epoxy ply reinforced with 0°/90° fabric was laid on the laminate front and back surfaces. In the aeronautical sector, it is common practice to apply one or more lightweight fiberglass fabric plies on the front and back surfaces of the laminate in order to limit the corrosion occurring as a result of the connection of materials of a different nature. The laminates were fabricated according to the procedure employed in the aeronautical industry, consisting of hand lay-up of prepreg plies, vacuum bag moulding, and curing cycle in autoclave. The curing cycle was controlled through the use of sensors within the autoclave, and it was performed according to the cycle suggested by the CYCOM 977–2 resin matrix producer. Heating was carried out up to 177 °C with speed equal to 2 °C/min, then, the laminate was held at 177 °C for 2 h, and eventually, a natural cooling in autoclave was accomplished.

Because of the fabrication process, the bag side laminate surface was irregular compared to the smooth mould side surface. To perform the drill-milling tests, specimens of 30 mm × 400 mm were cut from the original CFRP laminates.

### 3.2. Equipment

**Figure 2:**
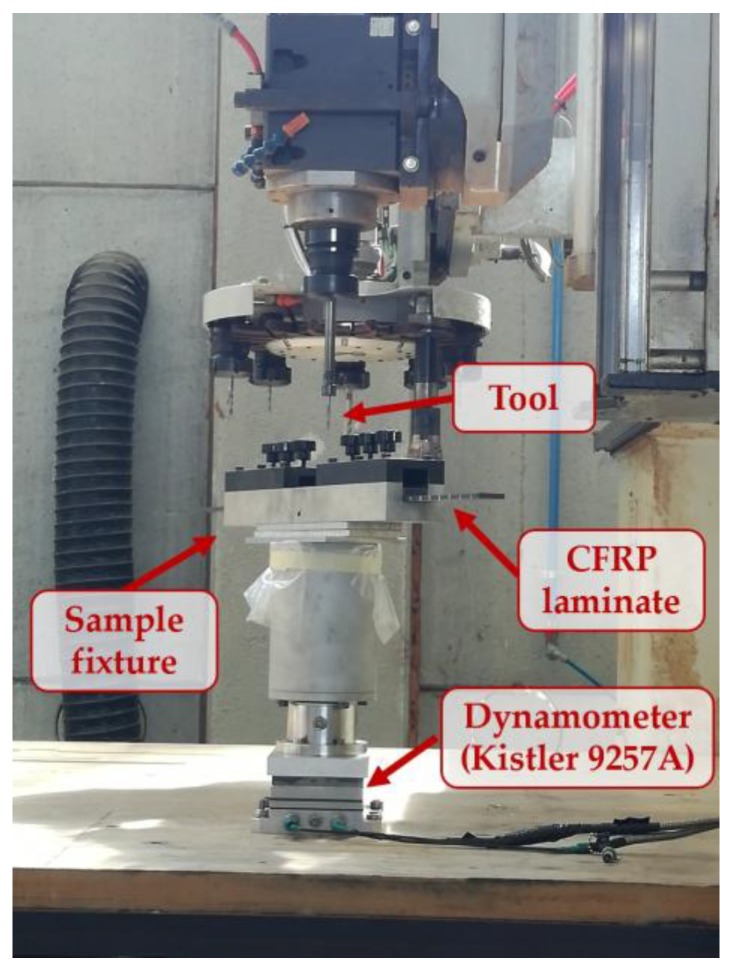
Equipment employed for the experimental drill-milling tests.

The drill-milling tests were performed under dry conditions on a five-axis Cosmec Conquest 3200 NC machining centre (Poggibonsi, Siena, Italy), equipped with a sensor system to detect the cutting force signals generated during the process (Figure 2). The signals of the thrust force, Fz, and radial force, Fx, were detected by a Kistler (Kistler Group, Winterthur, Switzerland) three-axis stationary dynamometer model 9257A, positioned under the workpiece fixture. The sensor system included a Kistler charge amplifier model 5007, and a National Instruments data acquisition board model 9239 (Austin, TX, USA) that digitized the Fx and Fz signals at 10 kS/s. 

The radial force can be resolved into two components acting along the *X* and *Y* dynamometer directions, respectively. As the process is symmetrical in the CFRP laminate plane, the values of the radial forces were similar, so that it was decided to report only the values recorded along the *X* direction.

**Figure 3:**
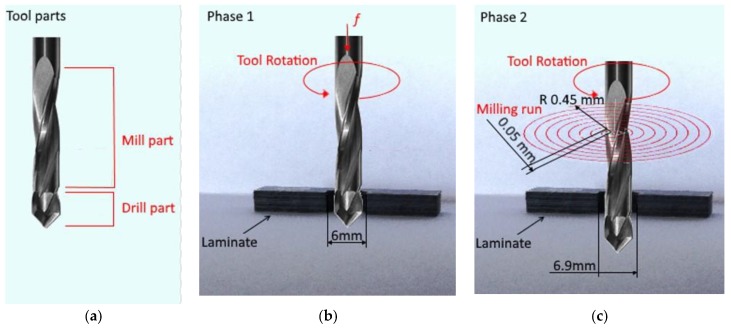
Representation of the following (**a**) tool parts and process phases; (**b**) drilling and (**c**) milling.

All of the tests were carried out using ISO CNC programming. According to the new drill-milling process, the first operation is the drilling of a pilot hole with a nominal diameter of 6 mm, followed by a peripheral milling operation. Specifically, once the pilot hole is realised, the tool follows a spiral milling path to increase the hole diameter by 0.10 mm per revolution, up to a hole diameter of 6.9 mm. Finally, three boring revolutions at constant diameter are executed. The process phases are shown in Figure 3.

In the experimental tests, the parameters selected for the drilling phase were chosen so as to limit the drilling-induced delamination damage to an extent that is lower than the radius of the material to be removed by the peripheral milling. In particular, the drilling speed was set to 10,000 rpm and the axial feed to 375 mm/min, while the milling speed was set to 14,000 rpm and the axial feed to 375 mm/min.

The drill-milling tests were conducted on 60 consecutive holes to analyse the process behaviour with a tool wear increase.

### 3.3. Tool

**Figure 4:**
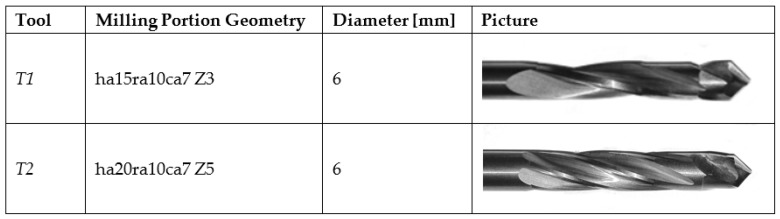
Comparison of the two newly developed drill-milling tools and milling portion geometry parameters (ha—helix angle; ra—rake angle; ca—clearance angle; Z—flutes number).

Two drill-milling tools characterised by different geometries were developed and employed for the experimental tests, to compare their performance. The characteristics and geometry of the two tools are shown in Figure 4. The first tool, T1, is made of tungsten carbide (WC) and has a 6 mm diameter and 100 mm length; the drilling portion is 8 mm long, with two cutting-edges, a 120° point angle, and a 20° helix angle; and the milling portion is 18 mm long, with three flutes, a 15° helix angle, a 10° rake angle, and a 7° clearance angle. The second tool, T2, is made of tungsten carbide (WC) and has a 6 mm diameter and 100 mm length; the drilling portion is 8 mm long, with two cutting-edges, a 120° point angle, and a 20° helix angle; and the milling portion is 18 mm long, with five flutes, a 20° helix angle, a 10° rake angle, and a 7° clearance angle.

### 3.4. Dimensional Analysis

In the aeronautical sector, very tight tolerance ranges are applied to the hole diameters [25]. In order to characterize the new drill-milling process, the produced hole diameters were measured with a digital calliper. The inner size of each hole was measured at a half laminate thickness along four directions (0°, ±45°, and 90°). The diameters were calculated as the arithmetic average of the measured values.

### 3.5. Surface Finish

To characterize the hole surface finish, roughness measurements were carried out using a stylus profilometer with tri-axial movement (Taylor–Hobson Form Talysurf-50, Taylor-Hobson, Leicester, UK) by dragging it along the x-axis a probe with a diamond tip radius of 2 μm. The roughness evaluations were performed through software code Ultra ver. 4.6.8, which allows for viewing and processing the data. 

To perform the measurements, the holes were sectioned; this was done every ten holes (number 1-10-20-30-40-50-60). The measurement length was 4 mm, with a probe travel speed of 0.5 mm/s, and the roughness tests were carried out according to standard UNI ISO 4288-2000 [26], a Gaussian filter with a 0.8 mm cut off was selected.

The roughness tests were performed three times for each hole, along three separate lines spaced by 0.05 mm.

The drilling of the CFRP laminates results in a series of micro-fibre fractures, fibre pull-outs, and matrix cracking; for this reason, the roughness parameter Ra (average) alone was not considered sufficient, and roughness parameters Rz (maximum peak to valley height) and RPc (peaks per mm count) were also evaluated [27].

## 4. Results and Discussion

### 4.1. Cutting Forces

**Figure 5:**
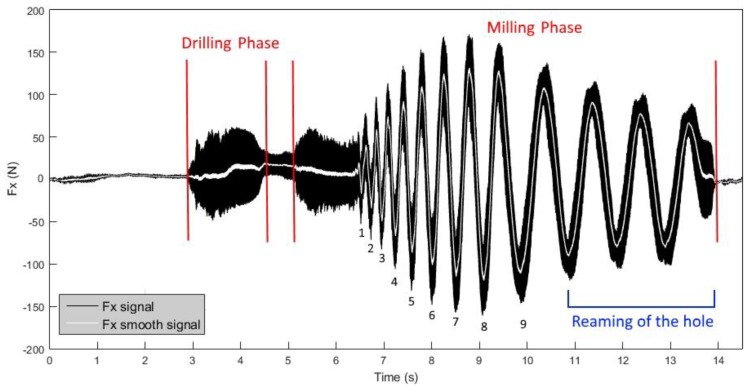
Signal of radial force, Fx, in the drill-milling process for tool T2.

**Figure 6:**
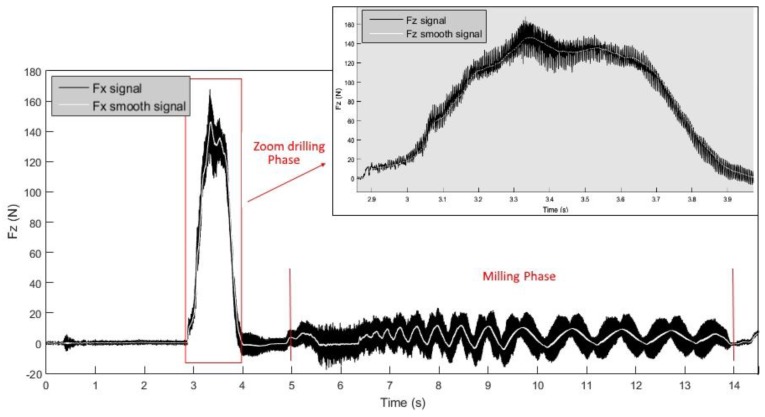
Signal of axial force, Fz, in the drill-milling process for tool T2.

The values and trends of the Fx and Fz cutting force signals were studied to analyse the cutting forces that develop during the drill-milling process. It was observed that Fz is predominant in the first phase (drilling process), while Fx is predominant in the second phase, where it is possible to observe the characteristic sinusoidal pattern of the peripheral milling process. To perform signal smoothing, the moving average method (with a window length of 200 samples, corresponding to 0.02 s) was applied to the Fx and Fz signals, as reported in Figure 5 and Figure 6, respectively. The goal of smoothing is to facilitate the extraction of the maximum values of the force signals, without taking into consideration high-frequency oscillations.

**Figure 7:**
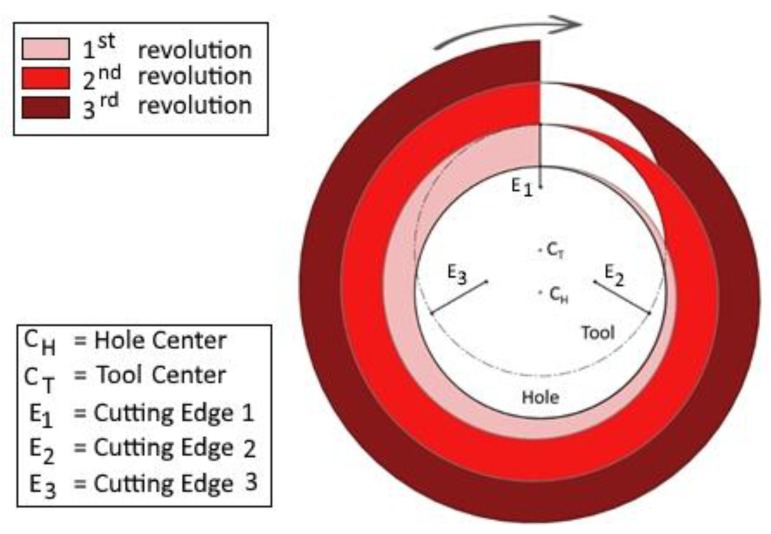
Scheme of the tool path during the drill-milling process. The removed material area increases at each revolution.

The Fx signals during the milling phase show a sinusoidal trajectory, where each sine wave represents a complete revolution of the tool inside the work-material. The size increase of the hole after each revolution is constant. The sine wave period increases, as the peripheral speed of the tool is constant, while the path to complete the whole revolution increases, as the radius becomes greater after each revolution. The volume of material removed also grows after, and during, each revolution as the radius of curvature increases (Figure 7). Because the work-material thickness is constant, the volume of material removed grows proportionally to the area increase, that is, with the square of the radius, according to a non-linear correlation. Moreover, the same considerations allow for stating that the amplitude of the sine wave also increases.

From Figure 7, it can be observed that, after a complete revolution, at the same polar coordinate of the previous revolution, the volume of material that the tool faces at the front cutting-edge is greater than for the previous revolution. Table 2 shows the values of the area of material removed after each revolution, calculated by means of a CAD software (Autocad 2017 version) tool. 

The growth of the area that the tool must cut leads to an increase of forces during the milling phase up to the eighth revolution, while the last revolution is characterized by a path that leads to the closure of the spiral and to a decrease of the cutting forces.

Then, there are three boring revolutions during which the sinusoidal waves display a decreasing amplitude, as shown in Figure 5, because the tool will follow the same path three times, removing less material at each revolution. 

Using the Pearson’s correlation coefficient, a strong correlation (>0.7) was found between the area of the material removed and the maximum value of the force in the *X* direction achieved after each spiral revolution. Specifically, a strong correlation was found for both tool T1 (0.86) and tool T2 (0.93).

**Figure 8:**
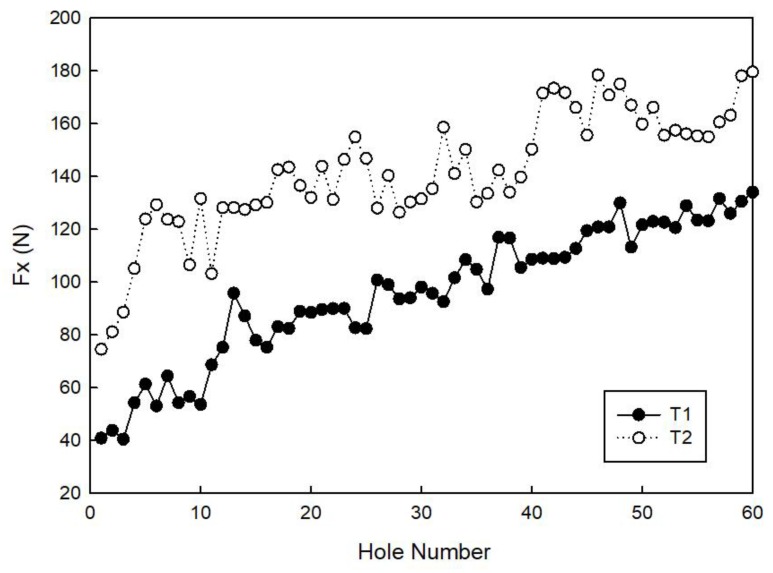
Maximum cutting force in the *X* direction, Fx_max_, measured for tools T1 and T2.

**Figure 9:**
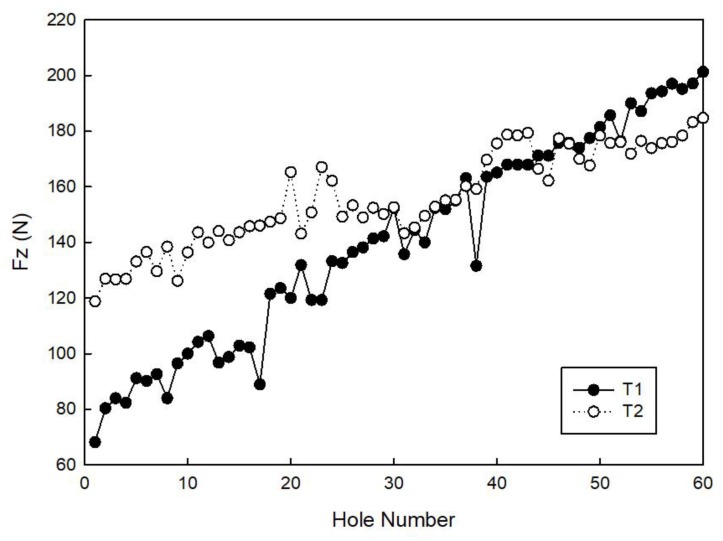
Maximum cutting force in the *Z* direction, Fz_max_, measured for tools T1 and T2.

Figure 8 and Figure 9 show the maximum Fx and Fz cutting force values measured for all of the 60 holes made during the experimental testing campaign with tools T1 and T2. In all of the cases, an increase of both the Fx and Fz cutting forces with an increasing hole number, that is, with tool wear progression, is visible. Moreover, it can be observed that, during the tests with tool T2, the recorded forces in both the *X* and *Z* directions are higher than the ones recorded with tool T1. Using tool T1, the maximum thrust force, Fz_max_, during the drilling phase is 201.27 N and the maximum radial force, Fx_max_, during the milling phase is 134.05 N (hole number 60). Using tool T2, the maximum thrust force, Fz_max_, during drilling is 188.41 N and the maximum radial force, Fx_max_, during milling is 179.57 N (hole number 60). It was verified that the values of Fz_max_ are quite similar to those found in the literature [8,12] for a traditional drilling process.

### 4.2. Metrological Analysis

With regards to the hole size, generally, hole diameter decreases with an increasing number of holes as a result of tool wear. Furthermore, this gradual decrease can be attributed also to the increase of tool deflection due to the growth of radial cutting force [9].

**Figure 10:**
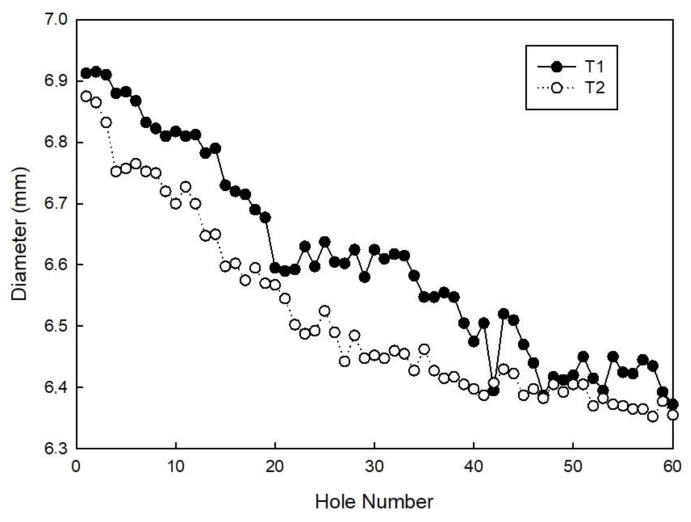
Hole diameter as a function of the number of holes.

This behaviour is confirmed by the measurements of the hole diameters, which always show a decreasing trend of the measured values for 60 consecutive holes made with the same parameters and tools, as shown in Figure 10. Diameter reduction is due to heavy tool wear during the drilling process, which is also caused by the decision to employ uncoated WC tools. This decision was made to verify the tool geometry and the cutting parameters independently of the tool material.

As shown in Figure 10, up until hole number 48, the tool T1 has a lower size reduction, whereas from hole number 48, both tools behave in a similar way.

**Figure 11:**
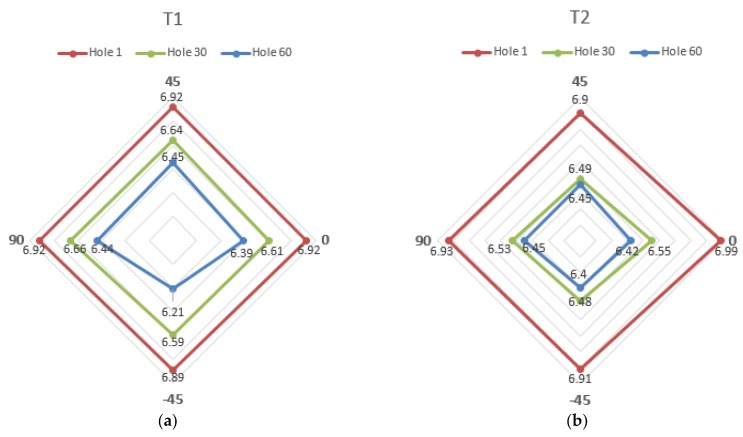
Hole diameter (mm) measured in four directions for holes number 1, number 30, and number 60, made by (**a**) tools T1 and (**b**) tool T2.

**Figure 12:**
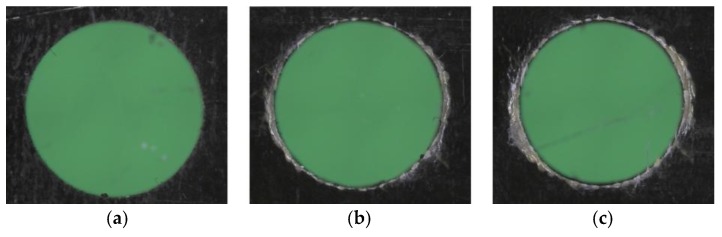
Pictures of (**a**) hole number 1, (**b**) hole number 30, and (**c**) hole number 60, made by tool T1.

**Figure 13:**
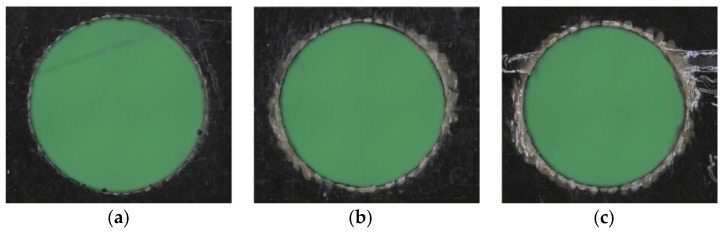
Pictures of (**a**) hole number 1, (**b**) hole number 30, and (**c**) hole number 60, made by tool T2.

With regards to the roundness of the CFRP holes, Figure 11 shows that the holes made by tool T1 keep their roundness up to hole number 30, and lose their roundness in one direction at hole number 60, whereas the holes made by tool T2 do not display any loss of roundness. Figure 12 and Figure 13 show holes number 1, number 30, and number 60 realized with tools T1 and T2, respectively. No presence of uncut fibers was verified in the holes, even in the case of high tool wear, contrarily to what often happens for traditional drilling [14,25]. This was true for both the T1 and T2 tools, as it can be observed in Figure 12c and Figure 13c.

### 4.3. Surface Roughness

**Figure 14:**
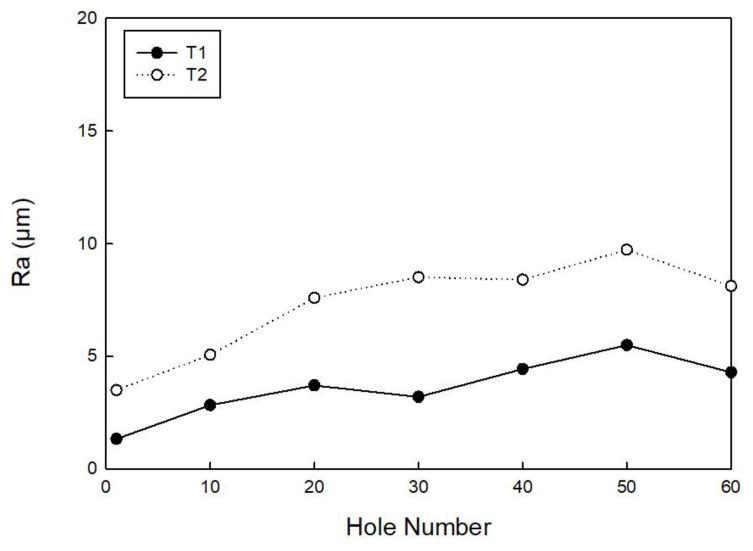
Ra measured for every ten holes made by tool T1 and tool T2.

Figure 14 reports the Ra values for tools T1 and T2, showing in both cases an increase with the increasing hole number. The holes made with tool T2 have internal surface Ra values greater than those made with tool T1. For the holes made with tool T1, the difference of Ra values between hole number 60 and number 1 is 2.96 µm, whereas for the holes made with tool T2, the difference of Ra values between hole number 60 and number 1 is 4.62 µm. As identical cutting conditions were employed with both tools, the Ra difference can be related to the different wear level of the tool cutting-edge [28,29].

**Figure 15:**
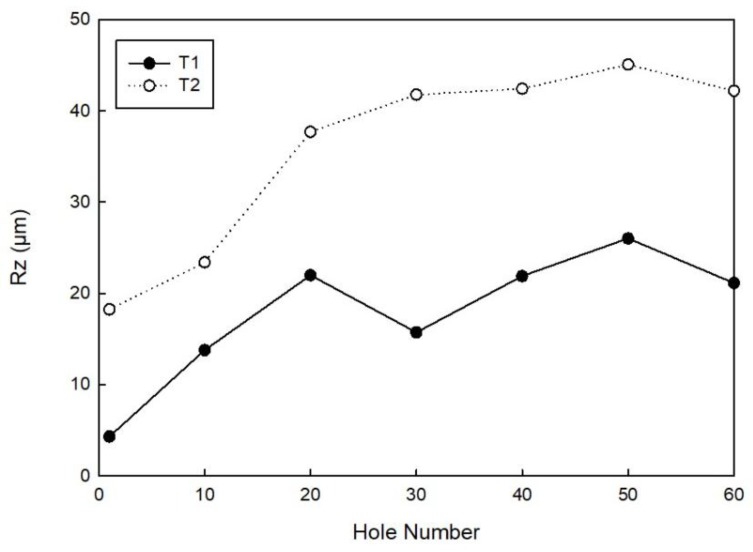
Rz measured for every ten holes made by tool T1 and tool T2.

Figure 15 reports the Rz values measured for every ten holes made by both tool T1 and tool T2. This roughness parameter demonstrates that, although the Ra values are slightly different, tool T1 responds with a better cut of the fibres. Taking into consideration hole number 1, the Rz values for the two tools differ by 13.92 μm, whereas for hole number 60, they differ by 21.07 μm, showing that tool T2 generates a severer fibre pull-out phenomenon.

**Figure 16:**
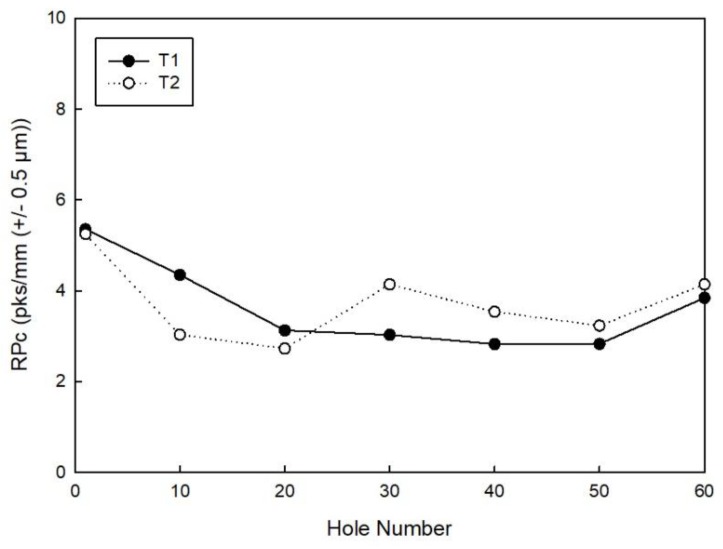
RPc measured for every ten holes made by tool T1 and tool T2.

Figure 16 reports the trend of the RPc parameter measured for every 10 holes. It can be observed that the number of peaks per mm is not high for both tools (only peaks exceeding ±0.5 µm were considered). This roughness parameter indicates that the surface finish of the hole is good, but there are gaps due to the fibre pull-out phenomenon, as observed in the literature on CFRP drilling [30,31]. 

## 5. Conclusions

This study presented an innovative hole-making process for CFRP laminates, where the hole is generated by a combination of drilling and peripheral milling processes. To develop this technique, innovative drill-milling tools were designed and realized, and experimental machining tests on CFRP laminates were performed using two different tool configurations, T1 and T2. During the drill-milling process, the thrust cutting force and the radial cutting force were detected through a sensor system to analyse the cutting force behaviour. The holes made with the two cutting tool configurations employing identical cutting conditions were characterized in terms of size, roundness, and surface finish. The following conclusions can be drawn from the obtained results:

- The maximum values of the thrust cutting force, Fz_max_, occurring during the drilling phase are consistent with those obtained in the experimental testing campaigns of traditional drilling.
- In terms of the cutting forces, the results obtained with tool T1 are better than those obtained with tool T2, as lower forces were recorded.
- In both tools, a rapid reduction in the diameter of the hole was observed, due to speedy tool wear growth, and also because the selected WC tools were uncoated.
- Tool T1 responded better in terms of a decrease in the hole diameter, and the use of a coated WC tool could lead to further improvement; it was also observed that the roundness of the hole is maintained with the increasing hole number.
- By comparing the measured roughness parameter values, the trends display a growing tendency with an increasing hole number in all of the cases. Moreover, the Ra roughness values are similar for both tools.
- The Rz roughness parameter values are better for tool T1. From this result, it can be inferred that tool T1 induces a lower fibre pull-out phenomenon in the CFRP.
- The RPc roughness parameter values indicate that the number of peaks per mm inside the CFRP is low, suggesting that the surface finish is good, except for the points where the voids caused by the pull-out phenomenon appear.

Overall, this process represents an interesting opportunity to reduce the delamination damage produced by traditional drilling and, although to a minor extent, by orbital drilling. The advantages of this new hole-making process are represented by the ease of programming, the absence of coolants, with benefits in terms of green technology, and the good surface finish, as shown by the roughness measurements, with particular reference to the RPc parameter. As a future development, the use of coated WC tools will be tested to further improve the surface finish in terms of all of the measured roughness parameters. Moreover, different path strategies will be tested in order to verify the potential benefits in terms of the hole quality and tool wear improvement.

## Figures and Tables

**Table 1:** Main mechanical properties of the unidirectional plies.

Property	Value
Young Modulus 0°	145 GPa
Tensile strength 0°	220 MPa
Young Modulus 90°	8.50 GPa
Tensile strength 90°	75 MPa

**Table 2:** Area of material removed after each revolution.

Revolution	Area of Material Removed (mm^2^)
1	0.46
2	0.86
3	0.88
4	0.90
5	0.93
6	0.96
7	1.00
8	1.04
